# On the Relevance of Using Open Wireless Sensor Networks in Environment Monitoring

**DOI:** 10.3390/s90604845

**Published:** 2009-06-19

**Authors:** Antoine B. Bagula, Gordon Inggs, Simon Scott, Marco Zennaro

**Affiliations:** 1 Department of Computer Science, University of Cape Town, 7707 Cape Town, South Africa; E-Mails: inggor001@uct.ac.za (G.I.); sctsim003@uct.ac.za (S.S.); 2 Telecommunication Systems Laboratory, The Royal Institute of Technology, Stockholm, Sweden and The Abdus Salam International Centre for Theretical Physics, Trieste, Italy; E-Mail: mzennaro@ictp.it

**Keywords:** wireless sensor networks, energy efficiency, open motes squidBee

## Abstract

This paper revisits the problem of the readiness for field deployments of wireless sensor networks by assessing the relevance of using Open Hardware and Software motes for environment monitoring. We propose a new prototype wireless sensor network that fine-tunes SquidBee motes to improve the life-time and sensing performance of an environment monitoring system that measures temperature, humidity and luminosity. Building upon two outdoor sensing scenarios, we evaluate the performance of the newly proposed energy-aware prototype solution in terms of link quality when expressed by the Received Signal Strength, Packet Loss and the battery lifetime. The experimental results reveal the relevance of using the Open Hardware and Software motes when setting up outdoor wireless sensor networks.

## 1. Introduction

The technology used for environment monitoring is evolving from manual and semi-mechanical systems into new sensing platforms where wireless sensor networks endowed with new communication protocols such as the IEEE 802.15.4 [1] and ZigBee [2] are offering new and fascinating ways of connecting embedded systems to the environment by converting physical phenomena into an electronic response. Wireless Sensor Networks (WSNs) are a new wireless technology which is currently deployed in both civil and military applications to achieve different sensing functions. These include: environment observation, healthcare and medical monitoring, home security, machine failure diagnosis, chemical/biological detection, plant monitoring, battlefield surveillance and enemy tracking. WSNs [3] are deployed in large numbers of tiny sensor nodes, each node being regarded as a cheap computer that can perform sensing, computation and communication. The sensor nodes operate with low power battery to perform physical, chemical and biological sensing activities. The sensor nodes communicate wirelessly and are deployed in three forms: (1) Sensor nodes used to sense the information, (2) Relay nodes used as relay for the information sensed by other nodes and (3) Sink nodes acting as base station with higher energy to transmit the sensed information to a local or remote processing place. The sensor nodes used to perform sensing and relay functionalities are commonly referred to as motes.

A typical WSN deployment scenario consists of placing sensing devices into an environment to collect key physical parameters such as humidity, light intensity and temperature, and to communicate the results via an Ethernet, WiFI or GPRS network to a local or remote centre. At this centre, the data collected as sensor readings is processed and appropriate decisions are taken about the environment. The efficiency of such a WSN deployment scenario may be measured by: (1) the WSN readiness for field deployment which involves the link quality of the WSN and (2) the economic effiency in terms of cost of deployment. As deployed in current generation WSNs, sensor nodes are range-limited, unreliable, failure prone, deployed with frequently changing topology and power limited, resulting in limited computational and storage capacity. Furthermore, existing WSN solutions are still expensive and not tailored for community networks and require significant engineering efforts to fine-tune their designs for these communities.

It is predicted that by allowing communication between inanimate objects, WSNs combined with RFID technology will bring a third dimension to the the “*first mile of the next generation Internet*”. This will lead to a “*Web-of-Things*” where information will be accessed not only “*anywhere and anytime*” but also by “*anyone*” and using “*anything*”. However, designing sensor nodes that address the economic and engineering issues raised above is still a challenge that needs to be widely addressed by the research community. The deployment of WSN platforms in real-life situations is also still in its infancy.

### 1.1. Related Work

Table 1 taken from [4] classifies the three main categories of sensors based on field-readiness, scalability and cost. While scalability reveals if the sensors are small and inexpensive enough to scale up to many distributed systems, the field-readiness describes the sensor's engineering efficiency with relation to field deployment. As revealed by the sensor classification for a set of physical, chemical and biological sensors, the WSN technology is still expensive for large scale deployment in hundred or thousands of nodes. As depicted in Table 1, physical sensors are relatively cheaper than chemical sensors excepted for some sophisticated light sensors. However, it is expected that WSN economic issues will be solved with the advances of the sensor technologies and the adoption from the industry will boost standardisation and thus mass production and subsequent price reduction. In terms of the engineering efficiency, Table 1 reveals high field-readiness for most physical sensors and for a few number of chemical sensors while most chemical sensors lie in the medium and low levels and biological sensors have low field-readiness.

The works described in [5, 6] are related to ours in terms of field-readiness and deployment of wireless sensor networks. Reference [5] presents the design of a new wireless sensor node referred to as *GAIA Soil-Mote* for precision horticulture applications which use precision agricultural instruments based on the SDI-12 standard developed for intelligent sensory instruments to monitor environmental data. The *GAIA Soil-Mote* mote is built around a communication infrastructure that uses the IEEE 802.15.4 standard while its software implementation is based on TinyOS [7]. Using a two-phase methodology including (1) laboratory validation of the proposed hardware and software solution in terms of power consumption and autonomy and (2) implementation to monitor broccoli crop in Campo de Cartagena in south-east Spain, the sensor node was validated under real operating conditions which revealed a large potential market in the farming sector, especially for the development of precision agriculture applications. The SquidBee [8] motes used in our experimentation also rely on the 802.15.4 standard for communication but use a different operating system. Reference [6] reveals the need for high temperature sensors capable of operating in harsh environments for disaster prevention from structural or system functional failures due to increasing temperatures and building upon the limitations of most of the existing temperature sensors proposes a novel passive wireless temperature sensor, suitable for working in harsh environments for high temperature rotating component monitoring. The proposed prototype sensor calibrated successfully up to 235°C proved the concept of temperature sensing through passive wireless communication.

References [9] and [10] address the issues of energy consumption in wireless sensor networks. In [9], the issue of energy consumption is revisited through a state-of-the art technology review of both fields of energy storage and energy harvesting for sensor nodes and energy harvesting is discussed with reference to photovoltaics, temperature gradients, fluid flow, pressure variations and vibration harvesting. A survey on energy consumption presented by [10] provides information pertaining to energy consumption in Rockwell's WINS node and MEDUSA-II. The survey reveals for example that for WINS, tuning the radio receiver increases the power consumption from 383 mW to 752 mW while MEDUSA-II increases its power from 10 mW to 22 mW. The same survey also shows that using the transmitter increases the power consumption from 771 mW to 1081 mW for WINS and from 19 mW to 27 mW for MEDUSA-II. This suggest that since the power limitation is such a constraint on WSN, it is appropriate to perform significant amounts of data processing and computation while the receiver is in active state, in order to reduce the amount of radio communication.

Several solutions have been proposed by researchers and practitioners to address the WSN engineering issues. These include topology control using preemptive and proactive mechanisms, multi-path routing and node activity scheduling. Preemptive toplogy control solutions consist of designing WSN layouts that optimise coverage, WSN lifetime and/or economic gain in number of WSN nodes used. By revealing optimal ways of connecting sensors through resolution of an optimal placement problem, layout optimisation studies such as those provided in [11-14] and [15] may also reduce energy consumption. While [11, 12] are based on a heuristic solution using multi-objective evolutionary optimisation, the placement problem in [13] is formulated as an non-linear optimisation problem solved using a self-incremental algorithm that adds nodes one-at-a-time into the network in the most efficient identified way. In [14], the optimal placement of static sensors in a network is used to help an agent navigate in an area by using range measurements to the sensors to localise itself. Reference [15] considers a cluster-based placement of beacons and data loggers which act as cluster heads in the Cosumnes River Preserve, with the objective of determining the minimum number and placement of beacons and data loggers for wireless sensors deployed in the preserve. This is implemented by formulating an optimisation problem which is solved by integer linear program (ILP). Proactive topology control includes a family of WSN layout designs [16-22] which apply (1) link activity control mechanisms [17,19-22] to decide for each node of a WSN the number of neighbour nodes which could result in limited broadcasting and energy consumption, and (2) node activity control mechanisms [16-18] allowing efficient control of a sensor node output power to avoid interference and limit energy consumption.

Reference [17] classifies topology control mechanisms into link- and node-based activity control mechanisms and surveys these mechanisms in terms of protocols and algorithms. In [19], the k-Neighbour protocol is proposed as a fully distributed, asynchronous, and localised protocol that achieves topology control based on the principle of maintaining the number of neighbours of every node equal to or slightly below a specific value k. The approach enforces symmetry on the resulting communication graph, thereby easing the operation of higher layer protocols. The work proposed in [20] consists of efficiently self-organising a network hierarchy with specific assignment of roles (or tasks) to sensors based on their physical wireless connectivity and sensing characteristics. The paper extends the hierarchical connected dominating set (CDS) construction algorithm to develop a role-based hierarchical self organisation algorithm for wireless sensor networks. The resulting self-organised sensor network establishes a network-wide infrastructure consisting of a hierarchy of backbone nodes, and sensing zones that include sensor coordinators, and sensing collaborators (or sensing zone members). LEACH [21, 22] is one of the most popular WSN protocols which is derived from the topology control using link activity control. It is build around a hierarchy where a WSN is subdivised into clusters and the nodes of a WSN are divided into normal nodes and cluster-heads.

Reference [16] proposes a distributed topology control algorithm which calculates the per-node minimum transmission power so that (1) reachability between any two nodes is guaranteed to be the same as in the initial topology; and (2) nodal transmission power is minimised to cover the least number of surrounding nodes. Analysis and simulation results obtained on a network of heterogeneous wireless devices with different maximum transmission ranges demonstrate the correctness and effectiveness of the proposed algorithm. The proactive topology control problem is modelled and analysed in [18] where a model is presented for analysing the performance of transmission strategies in a multi-hop packet radio network where each station has adjustable transmission radius. Building upon three comparitive transmission strategies, the results show that the network can achieve better performance by suitably controlling the transmission range and reveals that one of the transmission strategies referred to as “transmitting to the nearest forward neighbor by using adjustable transmission power”, has desirable features in a high terminal density environment.

The works presented in [23, 26] are proactive methods where link and node disjoint multi-path routing algorithms traditionally deployed in fixed networks are applied to WSNs to achieve energy conservation and reliability maximisation. While [23] uses multi-path routing in delay and reliability constrained wireless sensor network settings to increase the likelihood of reliable data delivery by sending multiple copies of data along different paths and decrease the data delivery delays by sharing the data transmission delay among the different paths available from the source to the destination, the work presented in [24] proposes the braided multi-path routing model where the node disjointedness constraint is relaxed by considering alternative paths which are partially disjoint from the primary path. A braided multi-path model based on constrained random walks to achieve almost stateless multi-path routing on a grid network is proposed in [25]while [26] proposes a braided multi-path routing model for WSNs referred to as Multi-Constrained Multi-Path routing (MCMP) where packet delivery from nodes to the sink is achieved based on QoS constraints expressed in terms of reliability and delay. This model addresses the issue of multi-constrained QoS in wireless sensor networks taking into account the unpredictability of network topology and trying to minimise energy consumption.

Sensor nodes may fall into one of the following states: (1) *Sensing* where a sensing node monitors the source using an integrated sensor, digitises the information, processes it, and stores the data in its on-board buffer, data which will be eventually sent to the base station (2) *Relaying* where a relaying node receives data from other nodes and forwards it towards their destination (3) *Sleeping* where most of the device components are either shut down or work in low-power mode and (4) Dead where a node is no longer available to the sensor network since it has either used up its energy or has suffered vital damage. A sleeping node does not participate in either sensing or relaying though it “wakes up” from time to time and listens to the communication channel in order to answer requests from other nodes. Upon receiving a request, a state transition to “sensing” or “relaying” may occur. Once a node is dead, it cannot re-enter any other state. The work presented by [27] proposes an OS-directed power management technique to improve the energy efficiency of sensor nodes by shuting down devices when not needed and waking them up when necessary but using an embedded microoperating system to reduce node energy consumption that exploits both sleep state and active power management. This allows the implementation of a correct policy for sleep-state transitioning that avoids the overheads of storing processor state and turning off power during sleep-state transitioning.

Sensor activity scheduling [28-30] is another energy minimising mechanism where decisions are made on which sensors need to be in which states and for how long in order to meet a given network coverage requirement, and maximise the network lifetime. More precisely, the network lifetime is the period from the network setup to the time that the deployed network can not provide adequate coverage. In [28], activity scheduling is addressed as a solution for the lifetime maximisation problem in wireless sensor networks by scheduling sensor nodes to work alternatively through configuring some of them to an off-duty status that has lower energy consumption than the normal on-duty one. Reference [29] addresses the problem of scheduling sensor activities to maximise network lifetime while maintaining both discrete K-target coverage and network connectivity. While K-target coverage requires that each target should be simultaneously observed by at least K sensors, the authors propose a model that considers both coverage and connectivity issues to ensure that the data generated by the sensors will be transmitted to the sink node via single or multiple hop communications by solving the sensor activity scheduling and routing problems jointly. Reference [30] proposes and analyses protocols that can dynamically configure a network to achieve energy conservation in WSNs, by scheduling sleep intervals for extraneous nodes while the remaining nodes stay active to provide continous service, in a setting where the active nodes maintain both sensing coverage and network connectivity.

### 1.2. Contributions and Outline

By leading to a paradigm shift where the intelligence of thousands of researchers and practitionners is made available to perform tasks in a grid fashion, Open Source WSN solutions such as the open motes SquidBee [8] and [31] provide the potential to address WSN issues and thus revolutionise research and practice. However, most current generation Open Source hardware and software WSN technologies are designed as development platforms suitable for academic purposes, which need hardware and/or software fine-tuning to be ready for field deployment. This paper revisits the problem of wireless sensor network deployment to assess the relevance of using the Open motes SquidBee technology to achieve environment monitoring. The main contributions of this paper consist of: (1) fine-tuning existing SquidBee technology to build a WSN prototype solution that can achieve engineering efficiency and (2) assessing the readiness of the proposed solution for field deployments with a specific focus on the achievable range and life-time of a WSN. We propose a prototype wireless sensor network which is built around the following key features:

#### Fine-tuning SquidBee hardware

SquidBee motes were originally designed to be powered by a single 9 V cell which is only able to power the mote for a few hours before it dies. Though the battery life can be improved to approximately 72 hours by using two 9 V cells in parallel, two 9 V cells are large and cumbersome, and it is expensive to replace two batteries per mote after every test. Building upon the openness of the SquidBee technology, we applied adjustments to the SquidBee motes circuitery to allow similar lifetime with the use of reachargeable batteries.

#### Fine-tuning SquidBee software

A SquidBee mote includes a microcontroller and a XBee communication module which, when awake draws 70 mA, while sleeping only draws 14 mA. Furthermore, a SquidBee mote only needs to stay awake for 0.5 seconds at a time to be able to send a complete packet. Using methods borrowed from the activity scheduling approach, we fine-tuned the SquidBee software to implement wake-up/sleeping mode mechanisms and mote energy control with the expectation of energy saving. The results reveal high WSN lifetime improvements compared to non-tuned motes.

#### Link quality characterisation

The link quality is an important parameter that may determine the readiness for field deployment of a WSN technology by provinding insight on its engineering efficiency. It has been widely used in several studies as a performance index that integrates specific performance parameters such as the received signal strength (RSS), the packet loss indicator (PLI), and the battery lifetime duration (BLD) to assess the readiness of specific sensor network technologies for real-world deployments. We propose, for the first time, a thorough characterisation of the SquidBee link quality parameters.

The remainder of this paper is organised as follows. Section 2 presents the main features of the SquidBee platform and describes the system architecture and the main features of our proposed prototype WSN solution. Section 3 reports on the experimental results obtained from two outdoor sensing locations in Cape Town and compare our findings to the performance of a Sun SPOT platform. Our conclusions and directions for future work are presented in Section 4.

## 2. The Wireless Sensor Network Prototype

**Figure 1.**
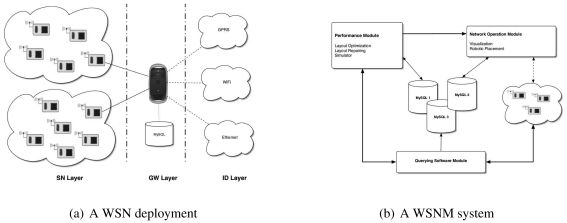
Wireless sensor networks.

A typical SquidBee WSN deployment scenario is depicted by Figure 1(a) where two WSNs are connected to a base station server that uses a database type of middleware to store and access data for local processing using Ethernet and remote dissemination using GPRS or WiFI. A service model of such deployment would lead to a layered view of a WSN which includes: (1) a sensor node (SN) layer (2) a gateway (GW) layer and (3) an information dissemination (ID) layer. The sensor node layer uses a set of three sensors (Light, Humidity, Temperature) to sense what is happening in the environment of the sensors and communicate this data to the base station where the information is passed to the base station for recording and further processing. The base station, also called the middleware layer connects the base station or sink node via a USB interface to a gateway which is a PC endowed with a set of database management systems. This layer's main services include: (1) data storage (2) data analysis and (3) local pre-processing and remote communication to make the information available for remote processing. The information dissemination layer is a layer where different communication technologies using different protocols and devices such as cellphones, PDA, and other borrowed from the fixed line communication technology such as ASDL or MPLS are used to disseminate the collected information to networks of users using a P2P, distributed or centralised dissemination model.

Building upon this service model, we designed for our prototype a wireless sensor network management (WSNM) system depicted by Figure 1(b), where the different software modules are used to deliver different services of the different layers of the layered view. The querying software module provides an interface for layer 1 and 2 services. It receives queries from the optimisation and middleware modules to get data from the WSN (link quality information) and readings from the sensing field (temperature, humidity,light), and then translates the data into user friendly input for these modules. The performance module provides an interface for layer 1 and 3 services by querying the status of the wireless sensor network via the querying software module, and by receiving data from the middleware module, it is able to perform optimisation and re-optimisation of the wireless sensor network. After layout optimisation, re-optimisation and/or simulation, this module sends the best network layout to the network configuaration module where visualisation is performed and node placement is performed in the WSN field through manual operation in user-friendly environments or using a robot in a human unfriendly environment. The middleware layer consists of a number of MySQL database systems which deliver an interface for layer 2 services and interfaces with: (1) the performance module (2) the visualisation module and (3) the network operation module. While the performance and network operation modules are important components upon which the efficiency of a wireless sensor network depends, the focus of this paper lies on the software that is used both on the sensor motes (the sensor node layer) and on the host system which was connected to the SquidBee base station (the gateway layer) and the hardware modifications performed on the motes to achieve readiness for field deployment.

### 2.1. Using Open Source SquidBee WSNs

Building upon the Open Source paradigm in both software and hardware, SquidBee motes are Open Wireless Sensors with freely downloadable source code and with fully available hardware description making possible for anyone to replicate the device without any special permission. SquidBee is an open hardware and software development platform that builds Wireless Sensor Networks (WSN) based on SquiBee motes. The SquidBee motes are easy to configure and allow WSN setup with minimal effort.

**Figure 2.**
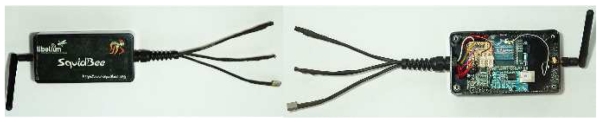
External and internal view of a SquidBee mote.

Figure 2 presents pictures of the exterior and interior of a SquidBee mote. The SquidBee mote consists of two separate circuit boards: the Arduino Diecimila board [35, 36] and the XBee shield board [37]. The Diecimila contains the microcontroller that runs the mote and the USB-to-serial converter chip that allows the microcontroller to be programmed over USB. The XBee shield simply contains the XBee RF module that is used for wireless communication between motes. The microcontroller used on the Diecimelia board is an Atmel ATmega168. It has a 16 MHz processor, 16K Bytes of flash memory for program storage and 1K Bytes of RAM. The microcontroller has six analogue input channels and twelve digital input channels, all of which are available on connectors on the board. The FTDI FT232RL USB-to-serial converter chip allows the microcontroller to communicate with a PC over a USB cable, as well as facilitating programming of the microcontroller over USB. The XBee shield board contains a MaxStream XBee RF module [38]. The RF module supports the IEEE 802.15.4 standard, as well as the ZigBee protocol. The module has a maximum transmit power of 0 dBm (software selectable), providing around 100 m line-of-sight communication outdoors. The receiver is sensitive down to -92 dBm. The XBee module allows communication over 16 direct sequence channels, each channel supporting 216 addresses. It supports unicast and multicast addressing, as well as point-to-point, point-to-multipoint and peer-to-peer topologies. Light, temperature and humidity sensors are provided with the SquidBee mote. These sensors connect to three of the analogue inputs on the Diecimila board as depicted in Table 2.

The total current consumption for the mote is 70 mA when active (i.e. when transmitting or receiving) and 14 mA when in sleep mode. The motes are programmed in language similar to Wiring, using the Arduino programming environment. The firmware is loaded onto the motes ATmega168 microcontroller via the USB connection. The firmware controls the transfer of packets between the microcontroller and the XBee module. The firmware also coordinates the reading of the sensors and any processing that must be performed on the data before it is transmitted.

### 2.2. Fine-Tuning SquidBee WSNs

Due to the open nature of SquidBee, the motes can be fine-tuned to establish many different WSN configurations. These include software and hardware configurations.

#### 2.2.1. Software Configuration: Fine-Tuning the SquidBee Software

Broadly the software used in our prototype had to: (1) facilitate the control of the SquidBee Sensors in terms of gathering environmental data and conserving battery life; (2) coordinate communication between the SquidBee Sensors and the SquidBee base station, which in turn needed to communicate with the host system connected to it and (3) provide access to the data gathered by the Sensors and Communicated to the base station. This functionality was achieved using software running on the SquidBee Sensors, written in the Ardunio programming language, and a middleware framework written in Python and making use of MySQL database software [39] running on the host system. This software is described below.

##### The power saving software

The software running on the SquidBee Sensor was a modified form of the Power Saving Code written and published by Marcos Yarza and David Gascon [40] of the Wireless Sensor Network Research Group. The code allowed for the SquidBee Sensor Motes, which includes the Ardunio Microcontroller and the XBee Wireless Shield to be powered down when not in use, greatly reducing current consumed, and hence conserving battery life. The code allows a periodic transmission of sensed data values, with a minimum power down period between samples of 5 seconds and a maximum period of 245 seconds. Unless otherwise stated the sampling period was 240 seconds. The original code required modifications to the SquidBee Sensor Mote hardware as described below in the Hardware Configuration subsection. It also required the reprogramming of the XBee Wireless Module used. The main modification of the code was to sense and transmit an additional value from one of the Analog to Digital Converter Channels, being the battery voltage level. A further modification of the code was that when a certain battery threshold was reached, the sensor was shut down completely, in order to protect the specific batteries that were used in these experiments. Additionally the XBee Wireless Module was programmed to transmit at 0 dBm (1 mW) in all the experimental trials, unless otherwise stated. Each Sensor was placed on a different channel, to reduce the chance of interference between different Sensors.

##### The Middleware Software

Middleware [41, 42] plays an important role in Wireless Sensor Network Research and as such the Middleware approach used for these experiments is discussed below. The approach is an attempt at a database abstraction, in which the sensor network is abstracted as a database for the end user. In order to achieve this, an actual rational database was used. The function of middleware software, which ran solely on the host system connected to the SquidBee base station, was to translate the data received from the SquidBee base station, and to insert the received data into the database. In order to achieve this received data was wrapped into Reading objects and passed from the base station Interface to the Database Interface by the Control Script. Thus all of the data received from the SquidBee Sensors by the SquidBee base station are inserted into a rational database of a general structure, allowing for the data to be extracted from the database and analysed at a later point. While the middleware software was designed to run on Ubuntu Linux 7.04, it can be adapted to run under any operating system which supports Python and MySQL.

#### 2.2.2. Hardware Configuration: Fine Tuning the SquidBee Hardware

**Figure 3.**
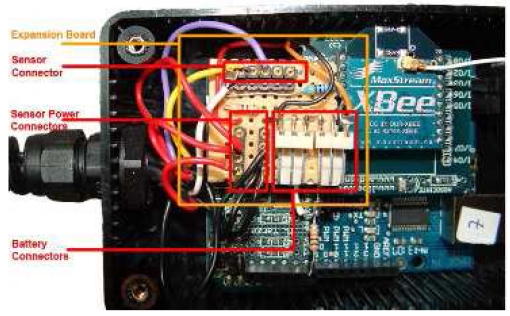
Photograph SquidBee mote with expansion board.

The hardware can also be expanded with relative ease, by adding additional circuitry. To improve the life-time of the SquidBee WSN, we achieved some fine-tuning to the hardware to allow the motes to (1) achieve energy conservation using a wake-up mechanism based on sleeping/wake-up modes and (2) use rechargeable batteries and a more convenient sensor connector. The SquidBee mote is designed to run off a single 9V alkaline cell. However to effect energy saving, the SquidBee motes can enter a sleep mode that requires a small hardware modification. For this investigation, we made three modifications to the SquidBee mote. First, we modified the motes to be powered by two rechargeable 3.7 V 1800 mAh lithium polymer cells connected in series. To allow the mote to enter sleep mode, two resistors were added to the XBee shield board, as described by [40]. With these batteries, the mote is able to run for approximately 100 hours before the battery voltage drops too low. Secondly, a further modification was done to allow ATmega168 microcontroller to monitor the battery voltage of the mote in real-time, using a spare analogue input on the Diecimila board. In order to prevent the voltage of either of the lithium polymer cells dropping too low and damaging the cell, the firmware on the microcontroller switched the mote off when the total battery voltage dropped below 7.42 volts. It was found that the mote was able to run for approximately 72 hours before switching off. Finally, a last modification to the motes was made to include additional connectors to make sensor connection more convenient. The above mentioned modifications were implemented on an expansion board that plugs on to the top of the XBee shield board. The expansion board is shown in Figure 3.

## 3. Performance Evaluation

This section describes the experimental environment used to evaluate the performance of the proposed Open Mote framework and discusses the experimental results obtained in an outdoor setting when using a star-based single-hop WSN composed of five motes and a base station to sense temperature, humidity and light intensity in two different sensing scenarios: 1) where the WSN is setup on top of the Leslie Commerce building of the university of Cape Town and 2) where the WSN is setup on top of the Groote Schuur hospital in Cape Town. Though many other aspects of the Open Mote Framework are currently being investigated, the focus of this paper lies on the evaluation of the link quality offered by the SquidBee technology expressed by the performance parameters described below.

### 3.1. Performance Parameters

This section details the performance parameters that were measured during the experiments to gauge the performance of the SquidBee Sensors.

#### Received Signal Strength Indicator (RSSI)

This indicator was measured in dBm, and indicates the strength of the electromagnetic signal received by the base station, relative to a milliwatt. The RSSI in these experiments was measured by the SquidBee base station operating in API mode. The minimum RSSI the XBeeTM module is stated as being capable of receiving is -92 dBm; however RSSIs of -94 dBm were recorded during these trials.

#### Packet Loss Indicator (PLI)

The Packets Lost Indicator used is a percentage indication of the number of packets not received versus the total transmitted. The PLI used in these experiments was an absolute value, measured in terms of the unique identifier of each data packet from each Sensor, in the application layer. The unique identifier was implemented as a count, and missing data packets were measured by missing values in the count. The 802.14.5 MAC (the communication protocol used) specifies 3 retries if there is an acknowledgement failure from the receiver. As such even if the packet is eventually received, this could have required several retransmissions. Evidence of these retransmissions could be sought in the relative Battery Lifetime Duration and RSSI of the sensors.

#### Battery Life Duration (BLD)

The Battery Lifetime was measured by comparing the time and date of the first and last transmission from each SquidBee Sensors. Additionally the voltage across the battery was sampled and also transmitted with the other sensor data. This was used to check that the battery had in fact reached its minimum voltage before the sensor shut itself down. The battery life duration gives a good indication of the amount of retransmits required for each data packet, as cumulative additional retransmits significantly decrease battery lifetime.

### 3.2. Overview of Experiments

Four experiments were performed in this investigation. The first two experiments investigated the effect of distance on the performance of the motes. For these two experiments, the five motes were positioned at distances ranging from 10 m to 130 m from the base station. In the first experiment, all motes were set to transmit at full power, while in the second experiment all motes transmitted at a reduced power level. For the third and fourth experiments, all motes were placed at the same distance from the base station. In the third experiment, the relationship between mote transmit power and RSSI, PLI and Battery Life Duration was explored by setting all motes to different transmit power levels. The fourth, and final experiment investigated the effect of different sampling rates on battery life.

### 3.3. Experimental Setting

**Figure 4.**
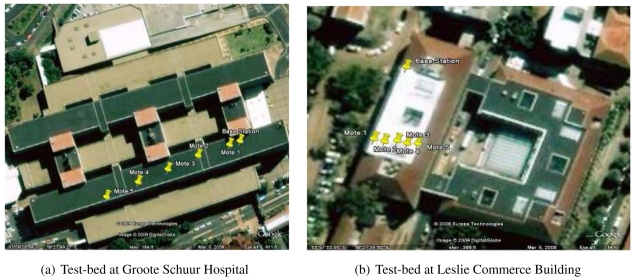
The two outdoor testbeds.

To ensure that the results of the experiment were as accurate as possible, a large open area was required that provided line-of-sight between all of the motes and the base station and that minimised the reflection of radio waves. Furthermore, the area needed to be as deserted as possible, as people walking about would interfere with the transmissions. At the end, two locations were chosen that matched the above criteria. The first was the roof of Groote Schuur Hospital in Cape Town, South Africa and the second the roof of the Leslie Commerce building of the University of Cape Town. Figure 4 shows the two test-beds, as well as the position of the base station and motes.

The distance of each mote from the base station, as well as each motes height above the ground, is shown in Table 3.

As one can see from the table above, the further a mote was from the base station, the higher it was positioned above the roof surface. This test-bed location was used for the first two experiments. For the third and fourth experiments, such a large distance between the motes and the base station was not required as no distance tests were being performed. Therefore a smaller test-bed was used. Here, experiments were performed on the roof of the Leslie Commerce Building, located on the Upper Campus of the University of Cape Town, in Cape Town, South Africa. Figure 4 (b) shows this test-bed, along with the positions of the base station and motes. In the second test-bed, all the motes were exactly 30 m from the base station and exactly 0.7 m above the roof surface. The base station was 0.4 m above the roof surface. For all four experiments, the motes and base station were mounted on thin poles to raise them to the required heights. The motes and base station were also placed in plastic bags to protect them from the rain. In all cases, the experiments ran until the batteries in all the motes died (usually around 72 hours). This meant that each experiment replicated field conditions, as it was performed over an extended period of time, in varying weather conditions. However, during none of the experiments did it rain.

#### Experiment 1: Distance Trial at Full Power

**Figure 5.**
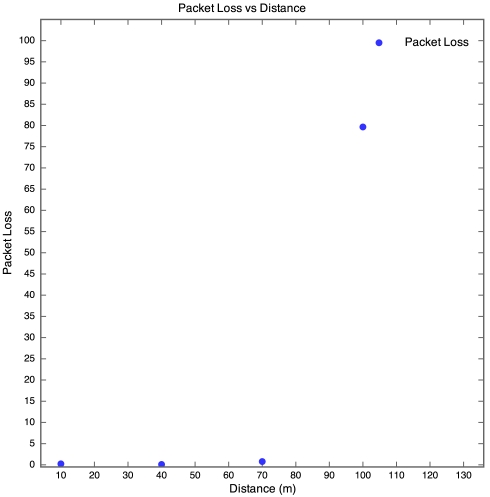
Packet loss vs Distance for Experiment 1.

**Figure 6.**
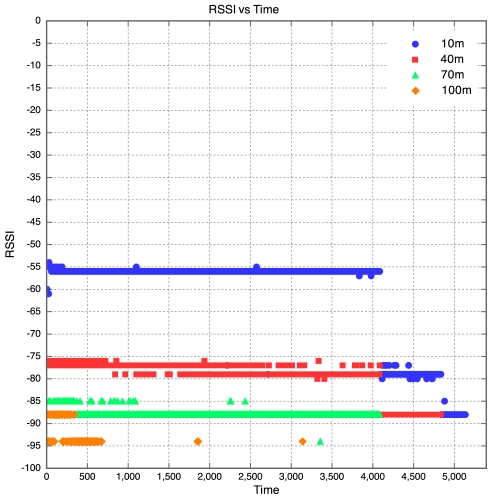
RSSI vs Time for Experiment 1.

The aim of this experiment was to determine the effect of distance between motes on RSSI, PLI and battery life duration (BLD). Five motes were placed at distances of 10 m, 40 m, 70 m, 100 m and 130 m from the base station, as described earlier. All motes were configured to transmit at maximum power (0 dBm). It was found that the mote at 130 m was too far from the base station for any of the packets to get through. As a result, no packets were received from the 130 m base station. Packets were received from the node at 100 m, but there was considerable packet loss. This is shown in Figure 5 which clearly reveals that above 90 m, the packet loss approaches 100% at an exponential rate. Therefore, as long as the motes are kept less than 90 m apart, the packet loss can be kept below 10%, giving good performance. The RSSI for each mote is plotted against time in Figure 6. Each point on the graph indicates the RSSI of the packet at that time for that mote. Therefore, missing points indicate lost packets.

The RSSI quite clearly decreases with an increase in distance, as would be expected. The receiver in the XBee RF module is sensitive down to -92 dBm. The mote at 100 m is sitting at -94 dBm, which seems to be the limit. As a result, many packets from the 100 m mote were lost. This also explains why no packets were received from the 130 m mote, as the RSSI of the packets from this mote would have been less than the RSSI of the packets from the 100 m mote, meaning the RSSI would have been well below the receiver sensitivity threshold of -92 dBm. Although the RSSI for each mote was fairly constant over time, there does seem to be a slight downward trend in the RSSI plots for the motes. This explains why it was found that the PLR for each mote increased with time. This is illustrated in Figure 6, where one can see that the majority of the packet losses for the 100 m mote occurred only after the 10 hour mark.

**Figure 7.**
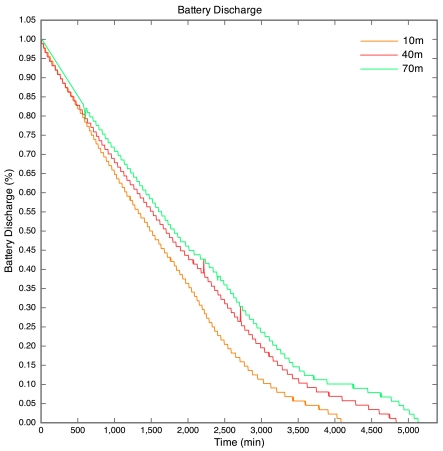
Battery discharge curves for Experiment 1.

The battery discharge curves for the motes are given in Figure 7. The discharge curve for the 100 m mote is incomplete, as all packets after the 43.5 hour point were lost. The discharge curves are non-linear due to the nature of lithium polymer batteries. The discharge curves have a similar shape to that of the typical discharge curve for the lithium polymer cells used in the motes.

**Figure 8.**
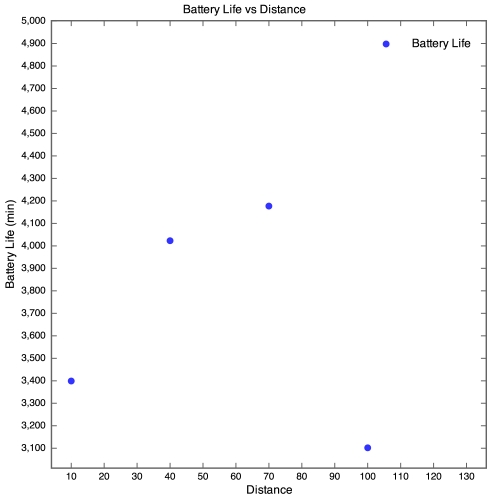
Battery lifetime for Experiment 1.

The discharge curve is steeper for motes closer to the base station than those further away. The result of this is that the battery life was longer for motes further away than for those nearer to the base station as depicted by Figure 8.

In Figure 8, no plot was made for the 130 m mote, as the battery discharge curve was incomplete and there was no way of knowing when the mote actually died. However, the discharge curve of the 100 m mote does seem to follow the discharge curve of the 40 m mote. It therefore seems realistic to estimate that the battery life of the 100 m mote would be similar to that of the 40 m mote, around 70 hours. Although no packets were received from the 130 m mote, the battery lifetime was determined manually by visually inspecting the mote on a regular basis and using the power and status LEDs as an indication of whether the mote was still running or if it had died. The normal behaviour of a WSN is for the furthest motes from the base station to have the shortest battery life as they perform more retransmissions than the closer motes. However, the SquidBee motes seemed to exhibit the opposite behaviour. This is probably due to the fact that the time taken to wake a SquidBee mote from sleep (0.5 seconds) is far longer than the time taken to retransmit a lost packet. Since the time taken to perform retransmissions is negligible and the SquidBee mote draws a constant amount of current when awake, regardless of whether it is actually transmitting or not, an increase in distance would not cause a significant decrease in battery life. We were unable to explain though why the battery life increased with distance, instead of remaining constant.

#### Experiment 2: Distance Trial at Reduced Power

**Figure 9.**
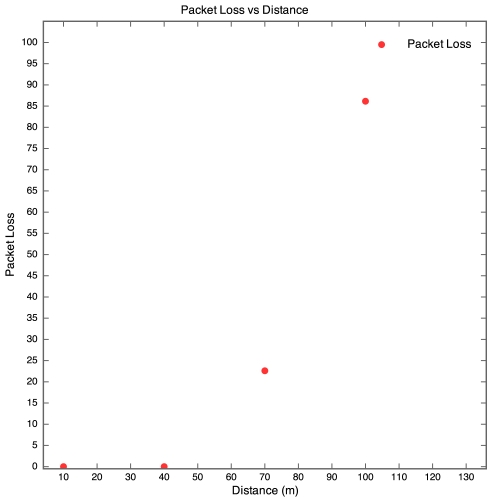
Packet Loss vs Distance for Experiment 2.

**Figure 10.**
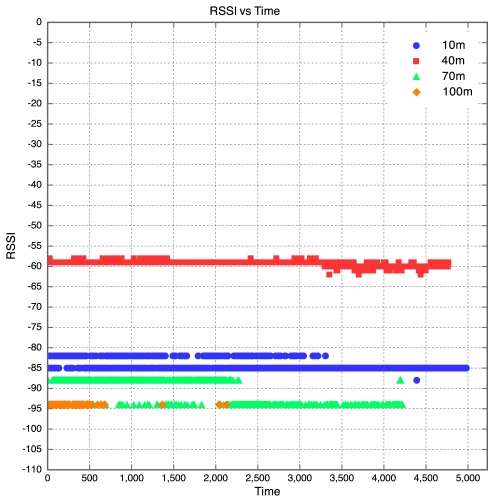
RSSI vs Time for Experiment 2.

Experiment 2 is a repeat of Experiment 1, with the motes now transmitting at the reduced power level of -4 dBm (as compared to 0 dBm in Experiment 1). At this reduced power level, we would expect to see higher packet losses, lower RSSI values and longer battery lifetimes than in Experiment 1. The packet losses for each mote are given in Figure 9. Note that no mote was placed at the 130 m point for this experiment, as we already knew from the previous experiment that all the packets from this mote would be lost. Again it can quite clearly be seen that packet loss increases with distance. The packet loss percentage for both the 70 m and 130 m motes increased significantly (0.8% to 23% and 80% to 86% respectively), when compared with Experiment 1. However, for the 10 m and 40 m motes, the packet loss did not increase at all when the transmit power was decreased. This indicates that if all the motes are less than 50 m apart, the transmit power can be decreased without increasing the packet losses. However, at this reduced power level, it seems that the motes do need to be within 60 m of each other to avoid excessive packet losses. Similarly, the RSSI for each mote decreased, although in many cases not by a significant amount. The exception was the 10 m mote which decreased from -56 dBm to -84 dBm. Strangely enough, the RSSI for the 40 m mote actually increased from -76 dBm to -59 dBm. This is because over such short distances interference from the environment has a bigger effect on the RSSI that signal attenuation. More importantly however, at the decreased transmit power level, neither the 10 m nor the 40 m mote lost any packets, and the RSSI only decreases slightly for the 70 m and 100 m motes as illustrated by Figure 10.

**Figure 11.**
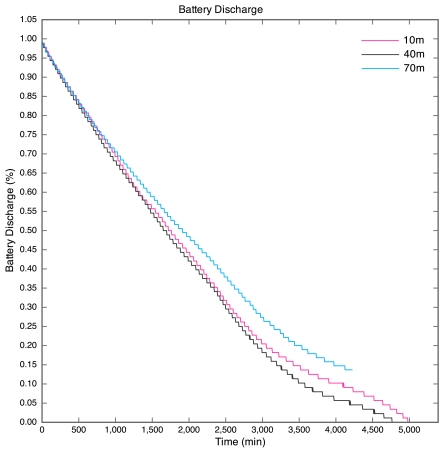
Battery discharge curves for Experiment 2.

**Figure 12.**
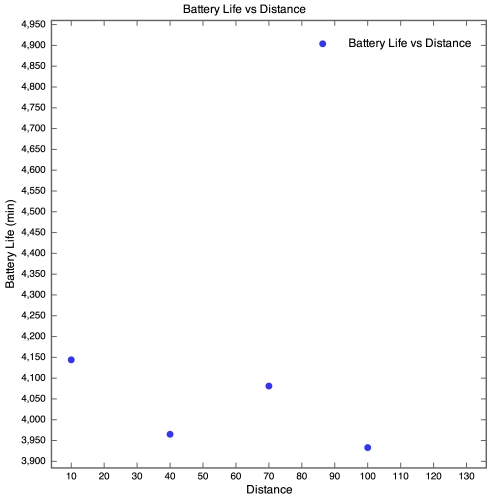
Battery Lifetime vs Distance for Experiment 2.

The discharge curves for the batteries for the motes are given in Figure 11. The discharge curve for the 70 m mote requires some investigation. It was found that the battery pack for the 70 m mote was charged to a higher voltage than the other battery packs. Therefore, the discharge curve of the 70 m mote is flatter than the curves of the other motes not because it was discharging at a much slower rate than the other motes, but because its initial battery voltage was much higher (0.1V higher). This also explains why the battery lifetime of the 70 m mote was much higher than the lifetime of the other motes, as depicted in Figure 12.

Just before the 60 hour mark, the pole mounting for the 70 m mote blew over. After this occurred, no more packets were received from the mote, which explains why the discharge curve stops just before 60 hours. However, based on the shape of the curve and data collected from other experiments, we estimated that the mote would have lasted 79 hours before the battery died. The discharge curve for the 100 m mote is incomplete as all packets after the 58.5 hour mark were lost, as the RSSI dropped too low. As a result, we were unable to determine the battery lifetime for the 100 m mote at the reduced transmit power.

Due to the fact that the 70 m motes battery was charged to a higher initial voltage than the batteries of the other motes and the fact that the discharge curve for the 100 m mote was incomplete, it is not possible to draw any definite conclusions regarding battery life of the motes from the second experiment.

#### Experiment 3: Different Transmit Power Level Trial

This trial was run on the Leslie Commerce test bed location. All of the SquidBee Sensors were placed 30 m from the base station, spaced radially. Each Sensor's XBee Wireless module was reprogrammed to use different transmit power levels, -10, -6, -2 and 0 dBm. The results are discussed below.

**Figure 13.**
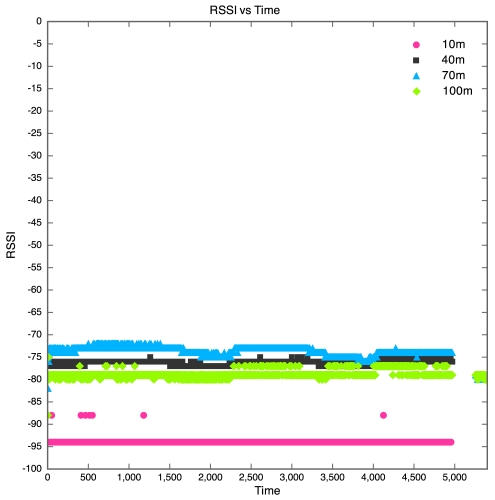
RSSI vs Time for different transmit powers for Experiment 3.

**Figure 14.**
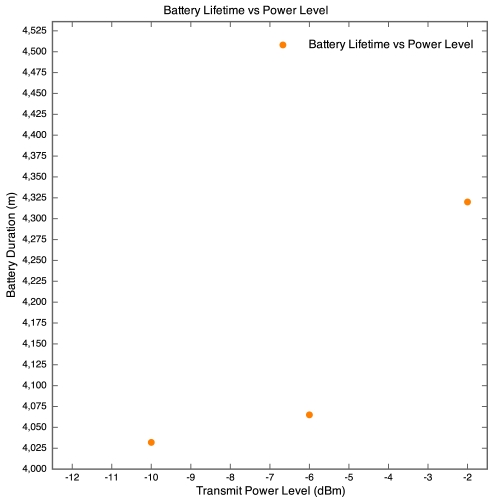
RSSI vs Time for different transmit powers for Experiment 3.

Figures 13 and 14 demonstrate RSSI vs Time of different transmit power levels. As expected, the Sensor set to the lowest transmit power (-10 dBm) exhibited the lowest RSSI values, with the values mostly at the maximum sensitivity for the SquidBee base station (-94 dBm). The remaining Sensors were within the -72 to -80 dBm band, which is a relatively small power difference between the signals. All of the Sensors in this trial demonstrated a similar Battery Lifetime duration, all within a range of 8 and a half hours. Significant was that the Battery Lifetime was proportional to Transmit Power, indicating that the data packets transmit at the higher power level were less prone to being retransmitted, and thus conserving battery life more significantly than any power saving induced by transmitting at a lower power level. It should be noted that the XBee Wireless Module Manual states current consumption and voltage draw to be constant, regardless of the transmit power level. The packet loss were less significant in this trial, as the Sensors transmitting at 0, -2 and -6 dBm lost no packets, while the Sensor transmitting at -10 dBm lost 11.9% of its data packets. This correlates with the RSSI values recorded, with the three higher powered sensors sensors clearly in a safe zone, with no packets dropped; while the lowest powered sensor was clearly transmitting at too low a power level, and hence was dropping packets.

#### Experiment 4: Different Sampling Rate Trial

Once again, this trial was run at the Leslie Commerce location, 30 m from the SquidBee base station, spaced radially. Each of the Sensor's onboard software was altered to use different sampling periods: 5, 10, 30, 60 and 240 Seconds. The results are discussed below.

The results of this trial are expected, with the exception of the Sensor running with a sampling period of 10 seconds. Although the Sensors are powered down during the sampling period, some power is still consumed, and as such the average current consumed by the different Sensors was within a set range. Barring the 10 second Sensor, all of the sensors exhibit a battery lifetime that is proportional to the length of their sampling period. It is interesting that the Sensors sampling every 30 and 60 seconds demonstrated a similar battery life, suggesting that the average currents consumed by the two Sensors lie on a flat part of the battery's discharge curve. It is likely that the 10 second sensor malfunctioned, as it consumed its Battery in a far quicker time than exhibited by any other Sensor, in any other trial. It is unlikely that the Sensor sampling every 5 seconds malfunctioned, as the malfunction would somehow have to have provided additional energy.

### 3.4. Link Quality Characterisation

Table 4 presents a summary of the experimental results presented above by comparing the engineering and economic parameters of the two open WSN platforms: Sun SPOT and SquidBee without and with fine-tuning (SquidBee+). The Sun SPOTS results are based on experiments conducted at KTH in Sweden on Sun SPOT motes as reported by [43] for similar outdoor condiftions. As can be seen, Sun SPOTs are much more powerful in processing and in memory while from the application point of view, SquidBees have the advantage of an external antenna connector and can be used with different batteries. An external antenna connector is useful to enhance the coverage of the radio, and different batteries can be choosed according to the application.

As revealed in Table 4, a WSN prototype using fine-tuned SquidBee technology (SquidBee+) can compete with a Sun SPOT technology in terms of some engineering efficiency parameters (life-time) but at reduced economic cost since SquidBee motes are cheaper and may obtained at additional reduced price of 20 euros when sold to be assembled by the purchaser.

## 4. Conclusions and Future Work

Building upon the openess of its development platform, this paper describes and evaluate the performance of a WSN prototype that fine-tunes the SquidBee technology to improve the readiness of SquidBee sensor networks for real-life deployments. Using the openness of the SquidBee hardware, we have fine-tuned the arduino hardware board to use rechargeable batteries which last longer than the few hours battery lifetime provided at pucrhase. We also adapted its software component to implement wake-up/sleeping modes to avoid unecessary energy consumption. We conducted testbed experiments in two outdoor settings in Cape Town which revealed the characteritics of the link quality of our porposed WSN solution. Comparative results based on previously conducted experiments on a Sun SPOT platform [43] reveal that a fine-tuned SquidBee technology can compete with the Sun SPOT technology in terms of engineering efficiency at reduced price.

There is room to extend the work presented in this paper in different directions. As new components of the first mile of the future Internet, 802.14.5 WSN based protocols will be inter-working with other wireless protocols such as WiFI, WiMax, WMPLS and fixed protocols as such ADSL to form the heterogeneous distributed backbone of the *Internet-of-the-thing*. The integration of these protocols into existing communication platforms while maintaining the scalability of the resulting systems is a direction for future work. Though WSN research and practice have been mostly directed towards single layer studies of link quality management or middleware designs, the interplay between these layers to achieve cross-layer optimization is another direction for future work. The WSN activity often results in massive datasets with hidden patterns which need to be discovered in order to take appropriate decision on the environment that is being monitored. Such discovery may be performed using methods borrowed from the artificial intelligence, Bayesian Belief Networks (BBNs) or other techniques. The integration of these techniques into the emerging WSN systems is another avenue for future research work. The focus of this paper was on a single hop routing model using a star topology to route the information collected by the SquidBee motes in the environment to the sink node using a direct wireless link. However, many practical deployments use multi-hop routing to overcome the range limitations of current WSN technolgies by allowing sensor nodes to play transit roles for the information collected by other nodes on its way to the sink node and gateway where further processing is performed. SquidBee and SunSPOTs use different mesh networking models. The comparison of the main features and efficiency of these two models have been reserved for future research work.

## Figures and Tables

**Figure 15.**
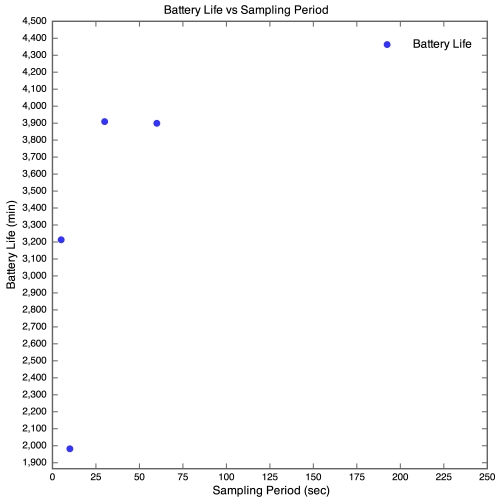
Different sampling periods for Experiment 4.

**Table 1.** Sensor classification.

Sensor Category	Parameter	Field-Readiness	Scalability	Cost (USD)

Physical	Temperature	High	High	50-100
	Moisture Content	High	High	100-500
	Flow rate, Flow velocity	High	Med-High	1,000-10,000
	Pressure	High	High	500-1,000
	Light Transmission (Turb)	High	High	800-2,000

Chemical	Dissolved Oxygen	High	High	800-2,000
	Electrical Conductivity	High	High	800-2,000
	pH	High	High	300-500
	Oxydation Reduction Potential	Medium	High	300-500
	Major Ionic Species (Cl-, Na+)	Low-Medium	High	500-800
	Nutrientsa (Nitrate, Ammonium)	Low-Medium	Low-High	500-3500
	Heavy metals	Low	Low	NA
	Small Organic Compounds	Low	Low	NA
	Large Organic Compounds	Low	Low	NA

Biological	Microorganisms	Low	Low	NA
	Biologically active contaminants	Low	Low	NA

**Table 2.** Sensor connectivity to the diecemila board.

Analogue Input Channel	Sensor

0	-
1	-
2	Temperature
3	Humidity
4	Light
5	Battery

**Table 3.** Experimental setting.

	Groote Schuur Hospital		Leslie Commerce Building	

Mote	Distance from base station	Height above roof	Distance from base station	Height above roof

base station	-	0.36 m	-	0.7 m
Mote 1	10 m	0.36 m	30 m	0.7 m
Mote 2	40 m	0.6 m	30 m	0.7 m
Mote 3	70 m	0.7 m	30 m	0.7 m
Mote 4	100 m	1.05 m	30 m	0.7 m
Mote 5	130 m	1.5 m	30 m	0.7 m

**Table 4.** Comparison of physical characteristics.

	**Sun SPOT**	**SquidBee**	**SquidBee+**
**Processor**	180 MHz 32-bit	16 MHz 8-bit	16 MHz 8-bit
**Memory**	512K RAM, 4M Flash	16K	16K
**Battery**	lithium-ion cell	—	lithium-ion cell
**Sensors**	acceleration, temperature, light	humidity, temperature, light	humidity, temperature, light
**Size**	41 × 23 × 70 mm	120 × 65 × 40 mm	120 × 65 × 40 mm
**Antenna Connector**	—	SMA	SMA
**Communication Protocol**	802.15.4	Zigbee	Zigbee
**Price**	630 euro for a kit of three	130 euro each	130 euro each
**Life time**	3 days before recharge	few hours	4 days before recharge
**Coverage**	100 m	100 m	130 m
**RSSI pattern**	similar to SquidBee	similar to Sun SPOTs	similar to Sun SPOTs
**PLI pattern**	similar to SquidBee	similar to Sun SPOTS	similar to Sun SPOTs
